# A Comparative Study of the Radical-scavenging Activity of the Phenolcarboxylic Acids Caffeic Acid, *p*-Coumaric Acid, Chlorogenic Acid and Ferulic Acid, With or Without 2-Mercaptoethanol, a Thiol, Using the Induction Period Method

**DOI:** 10.3390/molecules13102488

**Published:** 2008-10-15

**Authors:** Yoshinori Kadoma, Seiichiro Fujisawa

**Affiliations:** 1 Institute of Biomaterials and Bioengineering, Tokyo Medical and Dental University, Kanda-surugadai, Chiyoda-ku, Tokyo 101-0062 Japan; 2 Meikai University School of Dentistry, Sakado, Saitama 350-0283, Japan; E-mail: fujisawa33@nifty.com (S. F.)

**Keywords:** Radical-scavenging activity, Phenolcarboxylic acids, 2-Mercaptoethanol, BPO, AIBN, Induction period method, Prooxidant-antioxidant balance

## Abstract

Phenolcarboxylic acid antioxidants do not act *in vivo* as radical-scavengers in isolation, but rather together with GSH (glutathione), a coantioxidant, they constitute an intricate antioxidant network. Caffeic acid, *p*-coumaric acid, ferulic acid and chlorogenic acid with or without 2-mercaptoethanol (ME), as a substitute for GSH, was investigated by the induction period (IP) method for polymerization of methyl methacrylate (MMA) initiated by thermal decomposition of 2,2'-azobisisobutyronitrile (AIBN, a source of alkyl radicals, R**^.^**) and benzoyl peroxide (BPO, a source of peroxy radicals, PhCOO**^.^**) using differential scanning calorimetry (DSC). Upon PhCOO**^.^** radical scavenging, the stoichiometric factors (*n*, number of free radical trapped by one mole of antioxidant) for caffeic acid, ferulic acid, *p*-coumaric acid and chlorogenic acid were 2.4, 1.8, 1.7 and 0.9, whereas upon R**^.^** radical scavenging, the corresponding values were 1.3, 1.2, 1.0 and 0.8, respectively. Antioxidants with *n* values close to 2 suggest the stepwise formation of semiquinone radicals and quinones. By contrast, those with *n* values close to 1 suggest the formation of dimers after single-electron oxidation, possibly due to recombination of corresponding aryloxy radicals. The ratio of the rate constant of inhibition to that of propagation (k_inh_/k_p_) declined in the order chlorogenic acid > *p*-coumaric acid > ferulic acid > caffeic acid. The ratio of the observed IP for the phenolcarboxylic acid/2-mercapto-ethanol (ME) mixture (1:1 molar ratio) (A) to the calculated IP (the simple sum of phenol acid antioxidant and ME) (B) was investigated. Upon R**^.^** scavenging, the caffeic acid or *p*-coumaric acid/ME mixture was A/B>1, particularly the former was 1.2, suggesting a synergic effect. By contrast, upon PhCOO**^.^** scavenging, the corresponding mixture was A/B <1, particularly the latter was 0.7, suggesting an antagonistic effect. Upon both radicals scavenging, the A/B for the ferulic acid or chlorogenic acid/ME mixture was approximately 1. The reported beneficial antioxidant, anti-inflammatory and anticancer effects of caffeic acid and *p*-coumaric acid may be related to their prooxidant-antioxidant balance in the presence of GSH.

## Introduction

Phenolcarboxylic acids such as 3,4-dihydroxycinnamic acid (caffeic acid), 5-(3,4-dihydroxy-cinnamoyl)quinic acid (chlorogenic acid), *trans*-4-hydroxycinnamic acid (*p*-coumaric acid) and 4-hydroxy-3-methoxycinnamic acid (ferulic acid) exert beneficial effects on human health through prevention of degenerative pathologies such as cardiovascular diseases and cancer [1,2]. Caffeic acid and chlorogenic acid are two common coffee polyphenols. Ferulic acid was previously prepared from *Ferula foetida regel*, and recently also commercially from rice bran. Rice bran is well known to show antioxidant activity and is an absorber of ultraviolet radiation [3]. The antioxidant activity of olive phenols has been assessed using various tests such as inhibition of low-density lipoprotein oxidation [4], the lipid peroxidation inhibition capacity (LPIC) assay [5], 1,1-diphenyl-2-picrylhydrazyl (DPPH)-scavenging, and the inhibition of AAPH (2,2'-azobis(2-amidinopropane) dihydrochloride)-induced peroxidation of linoleic acid in sodium dodecyl sulphate micelles [6] and cupric ion reducing antioxidant capacity (CUPRAC) method [7]. We previously found that caffeic acid, chlorogenic acid and ferulic acid inhibited NO production in lipopolysaccharide (LPS)-stimulated mouse macrophage-like cells (RAW 264.7 cells) and scavenged various radicals such as superoxide anions and hydroxy radicals [8]. Also, we previously used differential scanning calorimetry (DSC) and the induction period method for polymerization of methyl methacrylate (MMA) initiated by thermal decomposition of 2,2'-azobisisobutyronitrile (AIBN) in order to investigate the radical scavenging activity of ferulic acid and related compounds under nearly anaerobic conditions, and found that ferulic acid scavenged radicals derived from AIBN [9]. This induction period method using the AIBN- and benzoyl peroxide (BPO)-MMA systems has proved to be reliable for evaluating the activity of phenolic compounds [10].

Antioxidants such as phenolcarboxylic acid do not act in isolation, but rather constitute an intricate network in the presence of coantioxidants such as glutathione (GSH), ascorbate, or other phenolic compounds such as vitamin E. The regeneration of polyphenols by synergistic reactions with ascorbate, GSH and vitamin E was previously investigated using enzymatic and non-enzymatic systems [11,12]. Also, synergistic effects have been previously observed in a rosmarinic acid/caffeic acid mixture, whereas antagonistic effects have been obtained with α-tocopherol/caffeic acid, (+)-catechin/caffeic acid, and caffeic acid/quercetin mixtures by 2,2'-azobis(2-amidinopropane) dihydrochloride-induced oxidation [13].

In the present study, we investigated the radical-scavenging activity of phenolcarboxylic acids, caffeic acid, *p*-coumaric acid, chlorogenic acid and ferulic acid, by determining the induction period (IP) for polymerization of MMA initiated by thermal decomposition of AIBN and BPO. Also, we investigated the radical-scavenging activity of a mixture of 2-mercaptoethanol (ME) and caffeic acid, *p*‑coumaric acid, chlorogenic acid or ferulic acid in equal molar proportions using the AIBN- and BPO-MMA systems. ME was used as a representative thiol because GSH cannot be studied in this system due to its limited solubility in MMA. ME is widely used for evaluating the biological role of GSH and cysteine.

## Results and Discussion

### Radical scavenging activities determined by the induction period method

**Figure 1:**
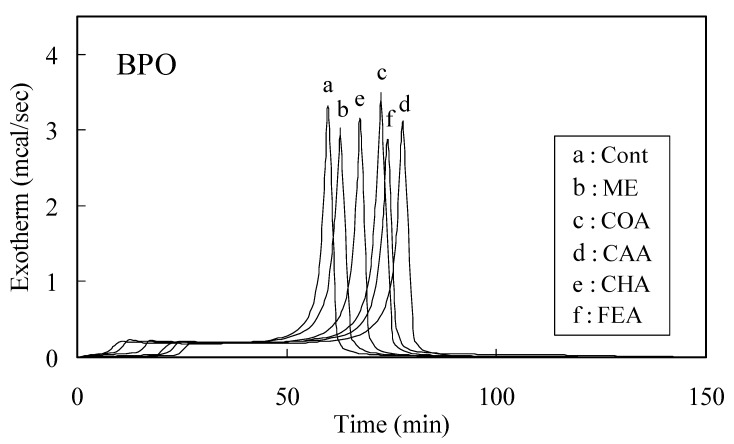
Exothermic curves for the polymerization of 9.4-mol L^-1^ MMA with 1.0 mol% BPO in the presence of 0.01 mol% additives; a: control, b: 2-mercaptoethanol (ME), c: *p*-coumaric acid (COA), d: caffeic acid (CAA), e: chlorogenic acid (CHA) and f: ferulic acid (FEA).

**Figure 2:**
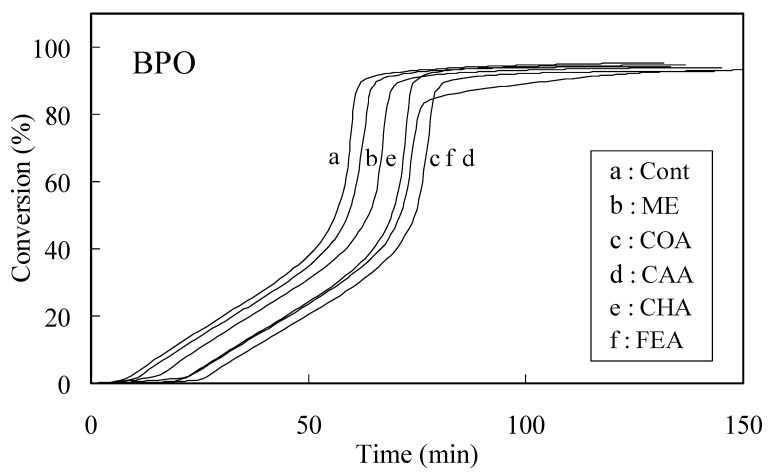
Time-conversion curves for the polymerization of MMA with AIBN in the presence of 0.01 mol% additives. Curves were obtained from findings shown in Figure 1. The abbreviation of compound names sees Figure 1.

The time-exothermic and time-conversion curves for caffeic acid, *p*-coumaric acid, chlorogenic acid, ferulic acid and ME in the BPO system are shown in Figure 1 and Figure 2, respectively. Figure 2 curves were organized by exothermic curves. The induction period (IP) and initial rate of MMA polymerization with an inhibitor (Rp_inh_) were determined from the curves shown in Figure 2. The IP for the BPO system declined in the order caffeic acid>ferulic acid>*p*-coumaric acid>chlorogenic acid>ME. Similarly, the order of IP for the AIBN system was similar to that for the BPO system. The *n* value (number of moles of radicals trapped by phenol calculated with respect to 1 mole of inhibitor moiety for phenol) was determined using Eq. (1). The *n* values upon PhCOO**^.^** scavenging were 2.4, 1.8, 1.7 and 0.9 for caffeic acid, ferulic acid, *p*-coumaric acid and chlorogenic acid, respectively, whereas the corresponding values upon R**^.^** scavenging were 1.3, 1.2, 1.0 and 0.8, respectively.

Caffeic acid, ferulic acid and *p*-coumaric acid upon PhCOO**^.^** scavenging gave *n* values close to two, whereas upon R**^.^** scavenging, all phenol acids showed *n* values close to 1. The *n* value for chlorogenic acid was approximately 1 for scavenging of both radicals. Antioxidants with *n* values of nearly 2 suggested the stepwise formation of semiquinone radicals and quinones; the phenoxyl radical is formed in the first step, and quinone in the second step.

When the *n* value is less than 2, dimerization may occur. When the *n* value is about 1, dimerization after single-electron oxidation occurs preferentially. Oxidation of caffeic acid, ferulic acid and *p*‑coumaric acid was previously reported to involve oligomerization, with dimer and trimer formation [5,6,13,14,15]. There was a clear difference in the relative *n* values between PhCOO**^.^** and R**^.^** radicals. Note: Although AIBN (R-N=N-R) in the presence of dioxygen delivers peroxyl radicals (ROO**^.^**), the present experiment was carried out under anaerobic conditions, and therefore AIBN delivered alkyl radicals (R**^.^**). 

**Table 1:** The ratio of the rate constant of inhibition to that of propagation (k_inh_/k_p_) for phenol carboxylic acids using the induction period method of polymerization of methyl methacrylate (MMA) initiated by thermal decomposition of 2,2'-azobisisobutyronitrile (AIBN) or benzoyl peroxide (BPO).

	Caffeic acid	*p*-Coumaric acid	Chlorogenic acid	Ferulic acid
AIBN	20.22	26.47	32.63	20.65
BPO	7.18	9.60	17.57	9.21

100 mM AIBN (or BPO); 9.4 mol L^-1^ MMA; 1 mM sample; at 70 °C. The procedures are described in the text. Means of three independent samples; Errors <7%.

The ROO**^.^** radical, and particularly the PhCOO**^.^** radical, is a strong electron/H-atom abstracting agent and is would probably react rapidly with phenolcarboxylic acid to form oxidation quinone intermediates. In the present study, the *n* value of antioxidants upon PhCOO**^.^** radical scavenging was greater than that upon R**^.^** radical scavenging. Also, the kinetic chain length of MMA (KCL: the ratio of the rate of propagation to the rate of initiation of initiator, Rp_con_/R_i_) for the PhCOO**^.^** radical was 614, whereas that for R**^.^** radical was 346, in the present study (Table 2). The former was approximately twice greater than that for the latter. These findings suggested that the reactivity of the PhCOO**^.^** radical with a phenolic acid is greater than that for R**^.^**. 

**Table 2:** Effects of the coantioxidant 2-mercaptoethanol (ME) on the induction period (IP) of *p*-coumaric acid, caffeic acid, chlorogenic acid or ferulic acid using the 2,2'-azobisisobutyronitrile (AIBN) or benzoyl peroxide (BPO)-methyl methacrylate (MMA) system.

Initiator	Phenols^a^	IP (min)			
		observed (A)	calculated (B)	B-A	A/B
AIBN	Caffeic acid	3.828			
AIBN	Caffeic acid +ME	4.893	4.135	-0.758	1.183**
AIBN	Chlorogenic acid	2.348			
AIBN	Chlorogenic acid + ME	2.694	2.655	-0.039	1.014
AIBN	*p*-Coumaric acid	2.966			
AIBN	*p*-Coumaric acid + ME	3.658	3.336	-0.322	1.097*
AIBN	Ferulic acid	3.757			
AIBN	Ferulic acid + ME	4.137	4.064	-0.073	1.018
AIBN	ME	0.307			
BPO	Caffeic acid	17.234			
BPO	Caffeic acid +ME	17.571	19.523	1.952	0.9*
BPO	Chlorogenic acid	6.371			
BPO	Chlorogenic acid + ME	7.887	8.236	0.349	0.958
BPO	*p*-Coumaric acid	12.841			
BPO	*p*-Coumaric acid + ME	10.867	14.77	3.903	0.736**
BPO	Ferulic acid	13.405			
BPO	Ferulic acid + ME	14.928	15.334	0.406	0.974
BPO	ME	1.929			

^a^ used at 1 mM each; MMA, 9.4 mol L^-1^; AIBN (or BPO), 100 mM; at 70 °C. The procedures are described in the text. The IP value of control for AIBN and BPO was 6.821 min and 3.880 min, respectively. IP_observed_=IP_found_-IP_control_. The Rp_inh_ is the initial polymerization-rate with antioxidant/ coantioxidant (ME) mixtures. The Rp_con_ is the initial polymerization-rate without any additives (control). The Rp_con_ value for AIBN and BPO was 2.074 x 10^-3^ mol L^-1^ sec^-1^ and 1.400 x 10^-3^ mol L^-1^ sec^-1^, respectively. Calculated IP (B) is the simple sum of antioxidant and coantioxidant. Standard error (n=3) <5%. *Significant difference vs A/B=1, p<0.05; **Significant difference vs A/B=1, p<0.01.

The antioxidant activity of phenolcarboxylic acids can also be expressed by k_inh_/k_p_. The k_inh_/k_p_ values were calculated using Eq. (5), and the results are shown in Table 1. For both the BPO and AIBN systems, k_inh_/k_p_ declined in the order chlorogenic acid>*p*-coumaric acid>ferulic acid>caffeic acid. The value for the BPO system was about half that for the AIBN system, possibly due to the R_i_ value of the initiator, as the R_i_ for AIBN was about double that for BPO. The k_inh_/k_p_ value was inversely proportional to the *n* value. Caffeic acid scavenged much more radicals than the other phenolic acids, and its k_inh_/k_p_ value was the smallest. This may have been related to the smaller BDE and E_1/2_ value [5,6,17]. 

### Mixtures of ME with caffeic acid, p-coumaric acid, chlorogenic acid or ferulic acid

To clarify the radical-scavenging activity of phenolcarboxylic acid in the presence of thiols such as GSH, we investigated the radical-scavenging activity of phenolcarboxylic acid/ME mixtures. The observed IP (A) and calculated IP (B) at molar ratios of 1:1 in the AIBN and BPO systems are shown in Table 2. In the AIBN system (R**^.^**, radical), the A of the caffeic acid/ME mixture and of the *p*-coumaric acid/ME mixture, particularly the former, were extended, compared with the corresponding B. Their ratio of A to B (A/B) was significantly greater than 1, suggesting a synergistic effect (A/B >1). The A/B for the caffeic acid/ME mixture was enhanced about 20%, whereas that for the *p*-coumaric acid/ME mixture was enhanced about 10%. Conversely, in the BPO system (PhCOO**^.^**), the A of the *p*-coumaric acid/ME and caffeic acid/ME mixture, particularly the former, was shortened, compared to the corresponding B. Their A/B ratio was significantly less than 1, suggesting an antagonistic effect (A/B <1). The A/B ratio of *p*-coumaric acid was reduced by 30%, whereas that of caffeic acid was reduced by 10%. Whether a synergistic or an antagonistic effect is observed for the mixtures derived from radical oxidation may depend on the chemical structure of the molecules and the possible formation of stable intermolecular complexes [13].

In general, regeneration between antioxidants occurs when the BDE of an antioxidant is lower, or at least similar to, that of other antioxidants [12]. The BDE (kcal/mol) of caffeic acid was previously reported to be 70.6 (1^st^; H-atom abstraction from 4-OH group) and 72.6 (2^nd^, H-atom abstraction from 3-OH group), whereas that of *p*-coumaric acid and ferulic acid was 75.2 and 73.1, respectively [6]. The BDE of aliphatic thiols such as ME was estimated to be 89±1 kcal/mol [18]. In the present study, the *n* value of caffeic acid and *p*-coumaric acid upon PhCOO**^.^** radical scavenging was about twice that upon R**^.^** radical scavenging. An antagonistic effect of these two compounds (A/B <1) occurred when their *n* value was close to 2, whereas a synergistic effect (A/B >1) occurred when the value was close to 1. Therefore, the prooxidant-antioxidant balance of these compounds may be controlled by their reaction activity with alkyl or peroxy radicals. However, the mechanism remains unclear.

We have previously reported the activities of caffeic acid, *p*-coumaric acid and ferulic acid for scavenging NO, O_2_**^-^** and **^.^**OH [8,9]. Some reports have documented their biological activities and physicochemical properties, including O-H bond dissociation enthalpy (BDE) and half-wave potential (E_1/2_), as summarized in Table 3. 

Caffeic acid preferentially inhibits LPS-stimulated NO production in RAW cells and also scavenges O_2_**^-^** and **^.^**OH [8]. Generally, BDE and E_1/2_ have been used as direct measures of the antioxidant properties of phenolic compounds, and the possible link between antioxidant activities and BDE or E_1/2_ has been reported previously [5,16]. Compounds with a smaller BDE or E_1/2_ are very active antioxidants [5]. Based on structure-activity relationships, the high radical-scavenging activity (NO, O_2_**^-^** and **^.^**OH) of caffeic acid may be related to its low BDE and/or E_1/2_. Also, the cytotoxicity of caffeic acid towards RAW cells is greater than that of ferulic acid [7], although the hydrophobicity (log P) of the two compounds is similar (log P=1.1) [5]. Also, the cytotoxicity of caffeic acid towards human submandibular gland carcinoma cells (HSG) is greater than that of ferulic acid [9]. Thus the cytotoxicity of phenolcarboxylic acids may not be dependent upon log P but upon radical-scavenging activity. 

**Table 3:** Some reported cytotoxicity, radical-scavenging activities (nitric oxide (NO); superoxide anion (O_2_^-^); hydroxy radical (**^.^**OH)) and physicochemical properties (phenolic O-H bond dissociation enthalpy, BDE; half-wave potential, E_1/2_) of phenolcarboxylic acids, caffeic acid, *p*-coumaric acid and ferulic acid.

Compounds	^a^ Cytotoxicity CC_50_, microM	Radical-scavenging activity				
		^b^NO	O_2_^-^	^.^OH	^e^BDE	^f^E_1/2_
		EC_50_, microM	^c^SOD U mg^-1^	^d^EC_50_, mM	kcal/mol	mV
Caffeic acid	13	0.5	305	0.77	70.8, 73.1*	531
*p*-Coumaric acid	>61	17	<0.1	1.07	75.2	942
Ferulic acid	>52	8.3	<0.1	3.22	73.10	753

^a^50% cytotoxic concentration (CC_50_) towards RAW 264.7 cells obtained from Ref [8]; ^b^the concentration that inhibits the NO production by 50% (EC_50_) in lipopolysaccharide (LPS)-stimulated RAW 264.7 cells [8]; ^c^superoxidase dismutase (SOD) unit (O_2_^-^) produced by the hypoxanthine and xanthine oxidase reaction [8]; ^d^50% inhibitory concentration of hydroxy radicals (**^.^**OH) produced by the Fenton reaction [8]; ^e^from Ref [6]; ^f^from Ref [16].

On the other hand, BDE is a strong indicator of radical scavenging because the cytotoxicity of phenolic compounds is related to their BDE value [17]. The high cytotoxicity of caffeic acid may be related to its low BDE, possibly resulting from the formation of quinones after two-electron oxidation. The DNA cleavage activity of caffeic acid was previously reported to be greater than that of ferulic acid or chlorogenic acid, possibly due to the large O_2_ consumption and H_2_O_2_ generation activity of caffeic acid [19]. The formation of *ortho*-quinone (*o*Q) from *ortho*-diphenol (caffeic acid) may be a key step in its DNA cleavage activity. Both electrolytic and enzymatic oxidation of caffeic acid also generates *o*Q. Oxidative conjugates of caffeic acid with GSH have been reported previously [20]. When GSH levels are low, *o*Q derived from caffeic acid may react with protein thiols, which can lead to toxicity. 

The radical-scavenging and biological activity and physicochemical activity of dihydroxycinnamic acids (caffeic acids and chlorogenic acids) with two hydroxy groups in *ortho* in aromatic ring have been previously reported. The DPPH**^.^**, ROO**^.^** and CUPRAC-radical-scavenging activity for caffeic acid is higher than that for chlorogenic acid [5,6,7]. The cytotoxicity of chlorogenic acid towards isolated rat hepatocytes is smaller than that of caffeic acid; the LD_50_ of the former and latter is 23 mM and 7 mM, respectively [20]. The small cytotoxicity of chlorogenic acid may be related to its hydrophobicity because chlorogenic acid (log P=0.6) is more hydrophilic than caffeic acid [20]. The order of peroxy radical scavenging activity of phenolcarboxylic acids has been measured as caffeic acid > chlorogenic acid > ferulic acid> *p*-coumaric acid, again entirely consistent with the results of the CUPRAC method [7]. Whereas, the order of radical-scavenging activity (the length of induction period) in the present study was measured as caffeic acid > *p*-coumaric acid > ferulic acid > chlorogenic acid. The order of ferulic acid and chlorogenic acid was different between the methods. Based on the stoichiometric factors, their *n* value in the present study was about 1, suggesting dimerization derived from parent monomers. Dimerization could be preferentially induced by the large intramolecular interaction between 2-methoxy and OH group in aromatic molecule in ferulic acid and that between OH groups in chlorogenic acid.

BDE as a physicochemical parameter can be approximated by the difference (ΔHf) in calculated heat of formation between parent phenolic molecule (Hfp) and its phenoxyl radical (Hfr), using the semiempirical PM3 method [5]. The order of ΔHf is estimated as *p*-coumaric acid > ferulic acid > chlorogenic acid > caffeic acid. The smaller ΔHf value is more active antioxidant [5]. The large radical-scavenging activity of caffeic acid may be related to its small ΔHf. 

Antioxidant capacity assays are broadly classified as electron transfer (ET)- and hydrogen atom transfer (HAT) based assays [7]. ET assays include the DPPH and CUPRAC methods. The majority of HAT assays are the kinetic radical-scavenging methods using azo-initiators. In the present study, we evaluated kinetically the radical-scavenging activity of the phenolcarboxylic acids using the AIBN and BPO-MMA systems; a HAT assay. Phenolcarboxylic acids scavenged radicals generated during MMA polymerization through a direct H-atom transfer process. The difference in k_inh_/k_p_ values and stoichiometric factors (*n*) for the phenolcarboxylic acids may be elucidated by their BDE value. The BDE of caffeic acid was the smallest [6] and this compound showed the largest *n* and the lowest k_inh_/k_p_ values. Caffeic acid, a potent antioxidant scavenged two radicals but its k_inh_/k_p_ value was small, judged by polymer chemistry.

*In vivo* experiments are too complex to be amendable to simple interaction, and therefore we carried out physicochemical studies using the IP method for the radical polymerization of MMA in the presence of antioxidants as a biomimetic model for scavenging of radicals produced *in vivo*. Such studies could help to explain the mechanisms by which phytophenols induce cancer cell apoptosis and act as chemopreventive agents.

## Conclusions

ME may control a balance of prooxidant-antioxidant for caffeic acid and *p*-coumaric acid during the induction period in polymerization of MMA initiated by AIBN and BPO. A synergistic or antagonistic effect of the caffeic acid or *p*-coumaric acid/ME mixture may suggest their potent chemopreventive activity of chronic diseases and cancers *in vivo*. 

## Experimental

### General

The following chemicals and reagents were obtained from the indicated companies: caffeic acid, chlorogenic acid, *p*-coumaric acid, 2-mercaptoethanol (ME), MMA (Tokyo Kasei Kogyo, Co., Ltd., Tokyo, Japan). Ferulic acid was obtained from Tsuno Food Industrial Co. Ltd., Wakayama, Japan. All reagents were of the highest purity available and were used without further purification. MMA was used with further purification. Each phenolcarboxylic acid and ME was solubilized in MMA at indicated concentrations and the mixture of equal parts of phenolcarboxylic acid and ME was prepared. AIBN and BPO (Wako Pure Chemical Industries Ltd. Japan) was recrystallized from chloroform and chloroform/methanol, respectively.

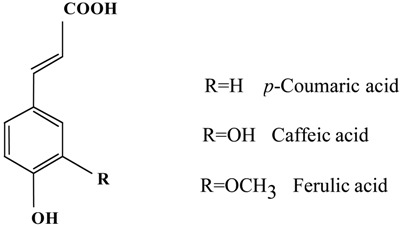



### DSC measurements

The induction period (IP) and initial rate of polymerization in the presence (Rp_inh_) or absence (Rp_con_) of a phenolcarboxylic acid and ME were determined by the method previously reported [10]. In brief, the experimental resin consisted of MMA and AIBN (or BPO) with or without additives. AIBN (or BPO) were added at 1.0 mol%, and the additives were used at 0 and 0.01 mol%. Approximately 10 µL of the experimental resin (MMA: 9.20-9.98 mg) was loaded into an aluminum sample container and sealed by applying pressure. The container was placed in a differential scanning calorimeter (model DSC 3100; MAC Science Co., Tokyo, Japan) kept at 70°C, and the thermal changes induced by polymerization were recorded for the appropriate periods. The heat due to polymerization of MMA was 13.0 kcal/mole in this experiment. The conversion of all samples, as calculated from DSC thermograms, was 92-96%. Polymerization curves were derived from DSC thermograms using the integrated heat evoked by the polymerization of MMA. Polymerization curves break when an inhibitor is consumed (Figure 2). These breaks are sharp and provide a reliable measure of the IP of the inhibitor. The presence of oxygen retards polymerization because oxygen reacts with MMA radicals activated by the initiator and then subsequently produces a non-radical product. Thus, polymerization of the control was slightly inhibited, even though the reaction was carried out in a sealed DSC pan, because the pan contained a small amount of oxygen since it had been sealed in air. Tangents were drawn to polymerization curves at an early stage in the run. The IP of test compounds was determined from the length of time between the zero point on the abscissa and the point of intersection of tangents drawn to the early stage of polymerization. The IP was calculated from the difference between the induction period of specimens and that of controls. The initial rates of polymerization in the absence (Rp_con_) and presence (Rp_inh_) of natural and synthetic antioxidants were calculated from the slope of the plots of the first linear line of the conversion rate of MMA polymerization (tangent drawn at the early polymerization stage).

### Rate of initiation

The induction period method was used to determine the rate of initiation (R_i_) due to the thermal decomposition of AIBN or BPO according to Eq. (1):

R_i_ = *n* [IH]_0_/[IP] (1)

where [IH]_0_ is the concentration of the inhibitor at time zero and [IP] is the induction period. 2,6-Di-*tert*-butyl-4-methoxyphenol (DTBM) was used to determine R_i_, since its stoichiometric factor, *n*, is known to be 2.00 [7]. In the case of [MMA] = 9.4 M and [AIBN or BPO] = 0.1 M at 70°C, the induction period method using DTBM gave the rate of initiation, R_i_, at 70°C. The R_i_ values of AIBN and BPO were 5.66 x 10^-6^ Ms^-1^ and 2.28 x 10^-6^ Ms^-1^, respectively [10].

### Measurement of stoichiometric factor (n)

The relative *n* value in Eq. (2) can be calculated from the induction period in the presence of inhibitors:

*n* = R_i_[IP]/[IH] (2)

where [IP] is the induction period in the presence of an inhibitor. The number of moles of peroxy radicals trapped by the antioxidant is calculated with respect to 1 mole of inhibitor moiety unit.

### Measurement of the inhibition rate constant (k_inh_)

When R_i_ is constant, i.e. when new chains are started at a constant rate, a steady-state treatment can be applied and the initial rate of polymerization of MMA is given by Eq. (3) [10]:

Rp_con_ = {k_p_ [MMA] R_i_^1/2^ }/(2k_t_)^1/2^ (3)

where MMA represents methyl methacrylate and k_p_ and k_t_ are the rate constants for chain propagation and termination, respectively. 

The k_p_/(2k_t_)^1/2^ rate of polymerization of MMA (9.4 M) by AIBN (1 mol%) and BPO (1 mol%) at 70°C was a constant value, 9.86 x 10^-2^ M^-1/2^ s^-1/2^. The Rp_inh_ rates are determined by Eq. (4):

Rp_inh_ = {k_p_ [MMA] R_i_} /{*n* k_inh_ [IH]} (4)

in which Rp_inh_ is the initial rate of inhibited polymerization, [MMA], *n*, [IH] and k_p_ are defined above, and k_inh_ is the rate constant for scavenging (inhibiting) of MMA radicals by an antioxidant. From Eq. (2) and Eq. (4), k_inh_/k_p_ can be calculated from Eq. (5):

k_inh_/k_p_ = [MMA]/{[IP] x [Rp_inh_]} (5)
